# Iranian Scientometrics; Dataset on universities, professors and articles

**DOI:** 10.1016/j.dib.2025.112285

**Published:** 2025-11-14

**Authors:** Mohmmad Shafei, Parsa Zahedi, Rojiar Pirmohamadiani

**Affiliations:** a Computer Engineering Graduate, University of Kurdistan, Pasdaran Blvd., Sanandaj, Kurdistan Province, I.R. 6617715175, Iran; b Department of Computer Engineering, Faculty of Engineering, University of Kurdistan, Pasdaran Blvd., Sanandaj, Kurdistan Province, I.R. 6617715175, Iran

**Keywords:** Google scholar, Web scraping, Scientometrics, Institutional research, Bibliometrics

## Abstract

This research introduces a comprehensive dataset of academic publications and professorial metrics from Iranian universities, systematically collected from Google Scholar using Python-based tools such as Selenium and BeautifulSoup, validated through expert review. articles.csv was kept raw except for exact duplicate removal, while a four-step Data Refinement Process (governmental affiliation, ≥ 100 citations, author-article verification, 2020–22 window) produced final_articles.csv for analysis. The dataset includes over 1.5 million records of articles scraped from various categories, providing detailed information on each article's title, citations, authorship details, and institutional affiliations, all curated through an intricate web scraping process. It spans multiple interlinked files with attributes including article metadata, professor profiles, and institutional details, We then applied a temporal filter (2020–2022) in conjunction with institution and author-level criteria, restricting to governmental universities and professors exceeding our citation threshold, and excluded records missing essential metadata (specifically, entries without titles or with removed/invalid Google Scholar links), yielding a focused cohort primed for downstream analytical pipelines. These attributes enable in-depth exploration of academic productivity, collaboration networks, and institutional performance across disciplines. The dataset provides a significant foundation for developing domain-specific models and theories related to academic impact and network analysis, with potential applications in social network analysis, trend identification in research disciplines, and benchmarking within the academic landscape. Additionally, it may support the development of machine learning and deep learning models for classifying research outputs and analysing scholarly trends, driving innovation in understanding academic ecosystems and informing data-driven strategies to enhance research excellence. This dataset is intended for use by scientometricians conducting citation and network analyses, university administrators performing institutional benchmarking, policymakers evaluating research strategies, and academic researchers exploring disciplinary trends. To remain fully compliant while capturing the complete corpus, we throttled requests far below Google Scholar’s implicit limits and deliberately distributed the crawl over a three-month window, trading speed for a policy-conformant, loss-minimised harvest. In short, we rate-limited requests, avoided circumvention, and did not bypass access controls; we acknowledge Google’s ToS restrict automated queries.

Specifications Table

**Table:** 

Subject	Data Mining and Statistical Analysis
Specific subject area	Research performance analysis using data mining and machine learning on Iranian universities and professors' publications
Type of data	Raw, Filtered
Data collection	Data for universities, professors, and articles was collected from Google Scholar. University profiles were retrieved from targeted URLs, while professor profiles and associated articles were scraped by navigating through linked Google Scholar university profiles.
Data source location	Google scholar
Data accessibility	Repository name: Mendeley Data Data identification number: https://doi.org/10.17632/r2xpddd84d.3 Direct URL to data: https://data.mendeley.com/datasets/r2xpddd84d/3 Shafei, Mohammad (2025), “Iranian Scientometrics; Dataset on Universities, Professors and Articles.”, Mendeley Data, V3, doi: 10.17632/r2xpddd84d.3
Related research article	None

## 1 Value of the Data

• **Benchmarking Academic Institutions (subject aware)**
  This dataset provides a robust foundation for comparing Iranian universities and their professors based on citation metrics, publication trends, and research output. In recent years, web-based indicators such as citation counts and online links have become an essential complement to traditional bibliometric measures [1]. It enables academic institutions to identify their strengths and weaknesses, supporting efforts to enhance global visibility and competitiveness. Construct **subject aware** academic research network graphs to rank researchers and universities based on their contributions and influence. This allows for benchmarking against national standards, and identifying areas for improvement.
• **Policy Development and Strategic Planning:**
  Insights from the dataset can aid policymakers in evaluating the effectiveness of governmental research initiatives, addressing regional disparities in academic contributions, and developing strategies to ensure equitable access to research opportunities nationwide. The comprehensive coverage of university profiles, professor metrics, and article classifications enables an in-depth analysis of research productivity, collaboration patterns, and the overall impact of Iranian academia. These findings can guide strategic decisions on resource allocation, faculty recruitment, and targeted initiatives to promote research excellence.

Additionally, investigating the role of the Ministry of Science, Research, and Technology policies in shaping the academic landscape is crucial. As [2] point out, despite the growing emphasis on internationalization in Iran's higher education policies, structural challenges and inconsistent implementation continue to hinder progress. Their findings underline the need for data-driven strategies, such as those enabled by our dataset, to guide policy development that can truly improve academic collaboration and institutional growth on a global scale [2]. By offering detailed metrics such as the h-index, i10-index, and citation counts, the dataset provides a robust foundation for objective evaluations of faculty and institutional performance. These insights can inform tenure decisions, optimize funding distribution, and support faculty development programs.

• **Trend Analysis in Research Disciplines:**
  Researchers can track emerging fields, interdisciplinary collaborations, and shifts in academic focus, guiding future investigations and funding priorities.
• **Social Network and Citation Analysis:**
  The dataset enables the exploration of academic networks through co-authorship and citation patterns, uncovering influential researchers, collaborative clusters, and the role of institutions in shaping Iran’s academic landscape. Such insights can help foster stronger research collaborations. By analyzing collaborative patterns within and between universities, the dataset highlights opportunities to strengthen research networks and promote interdisciplinary collaboration. Its structure is particularly suited for social network analysis, providing a detailed understanding of collaboration patterns and influence dynamics within the Iranian academic community. Additionally, the dataset can be utilized in regression models to identify factors driving research productivity and impact. The analysis also underscores the importance of local clusters in network formation within Iranian academia. This can reveal how geographic proximity, institutional affiliations, and other factors shape collaboration and facilitate knowledge diffusion. Still, co-authorship is an imperfect proxy for collaboration; interpret edges with these conceptual limits in mind [3].
• **Multi-layer analysis ready (U–P–A–S graph):**

Although our sources are not unique, the joinable design across Universities (U) → Professors (P) → Articles (A) plus Subjects (S) enables multiplex analyses: affiliation (U–P), authorship (P–A), co-authorship (P–P via A), and topical structure (A–S, aggregated to P–S and U–S). See Data Description → Derived multilayer representations for keys and joins.

• **Alternative to Traditional University Rankings:**

Older and traditional university ranking methods often rely on aggregated indicators and prestige-based metrics that can mask real academic performance. Many fail to normalize citation impact across disciplines or reflect the actual research productivity of faculty members. This dataset allows users to create their own data-driven ranking systems using normalized indicators such as citation counts, h-index, and i10-index. It supports more nuanced evaluations based on subject areas and collaboration networks, offering a customizable and transparent alternative. Notably, [4] proposed a ranking system based on the Crown indicator (CPP/FCSm), demonstrating that normalized metrics better capture research performance than raw publication counts. Their work revealed that smaller and newer Iranian universities outperformed older, more prestigious institutions when rankings were adjusted for field-based citation differences, a key insight that supports the design philosophy behind this dataset [4].

## 2 Background

Robust, open datasets that link universities, researchers, and article-level metadata are a prerequisite for reproducible national-scale scientometric work [5,6]. Google Scholar’s public university pages and author profiles offer a crawlable scaffold for constructing such a graph at scale [7]. Our contribution is an openly licensed, joinable corpus for Iran that integrates universities (U), professors (P), and articles (A), with optional subject tags (S), to support field-aware benchmarking, collaboration mapping, and reproducible downstream analyses [8,9].

The need for a consolidated, Iran-focused corpus is practical as well as methodological. Article-count growth has outpaced the availability of auditable, machine-readable linkages among institutions, scholars, and outputs; relevant records are scattered across individual Scholar profiles and ministry reports with inconsistent metadata. A single, structured release that (i) preserves a raw provenance layer and (ii) provides a worked, policy-oriented derivative enables both verification and reuse by different audiences. Additionally, national reports and indicators underscore the policy relevance of reliable, open evidence: Iran’s R&D context and quality concerns have been widely discussed, motivating transparent datasets that permit replication and auditing [[10], [11], [12], [13], [14]].

This design is aligned with Data in Brief’s objective to support secondary analysis. The dataset is intended for scientometricians (e.g., constructing co-authorship or field-normalized indicators), university administrators (institutional benchmarking and dashboards), and policymakers (evidence for funding or evaluation frameworks) [[12], [13], [14]]. To maximize usability, we (a) document column-level schemas and null rates for each file; (b) detail the harvest workflow and throttling strategy (Selenium + BeautifulSoup, conservative pacing) to clarify provenance and ethical considerations; and (c) expose the U–P–A–S joins for multi-layer analyses without prescribing a single analytical pipeline.

SCImago’s 2024 data show that about 40 % of Iran’s Scopus-indexed research is in engineering and technology fields and 34 % in medical and life sciences, while Arts & Humanities and Social Sciences together account for only about 6 %, one-third of the global average. This focus on low-citation disciplines, along with limited research funding—GERD at 0.73 % of GDP versus the OECD’s 2.70 %—and sanctions restricting international collaboration, helps explain Iran’s modest field-normalized impact despite high output. The new openly licensed dataset introduced here links Iranian universities, scholars, and 1.5 million articles to enable more detailed, field-based national benchmarking than global scientometric tools currently provide.

## 3 Data Description

**Fig. 1:**
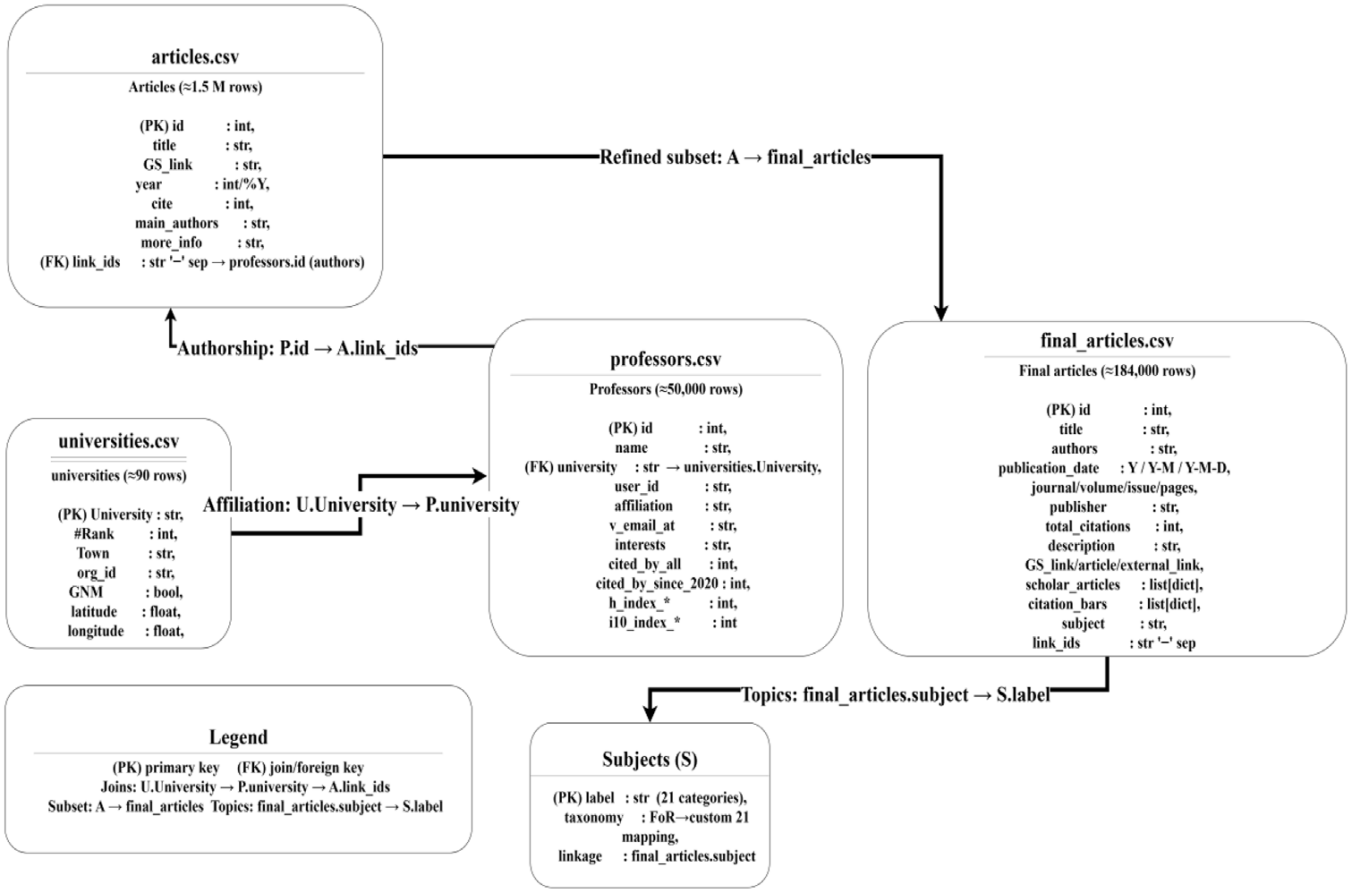
Data package overview & entity–relationship (ER) diagram (U–P–A–S).

This dataset offers a unique and comprehensive view of the academic landscape in Iran, covering universities, professors, and their research outputs. It’s worth mentioning that the professors’ dataset and file was scraped in 2025 again to be up to data and more accurate. Our datasets are Scraped from Google Scholar, it includes detailed profiles of ∼50,000 professors, ∼1.5 million articles, and rankings and metadata for 90 universities. The dataset provides rich metadata, encompassing national rankings, citation metrics such as h-index and i10-index, and detailed yearly citation trends, making it ideal for performance evaluations at both institutional and individual levels. These metrics are closely aligned with those used by the AD Scientific Index, which evaluates the research performance of universities based on aggregated citation data from individual scholars, offering a transparent and granular approach to academic benchmarking [15]. To orient users before the file-level details, Fig. 1. Data package overview & entity–relationship (ER) diagram (U–P–A–S) summarizes the data package and the entity–relationship (ER) model that governs joins among universities (U), professors (P), articles (A), and subjects (S).

Geographic data included in the dataset enables spatial analysis of universities across Iran, offering insights into regional distributions of academic excellence. Additionally, the temporal citation data supports longitudinal studies of research output and its evolution over time. The professor and article datasets are interlinked, allowing for detailed network analyses of collaboration patterns among researchers and institutions.

**Fig. 2:**
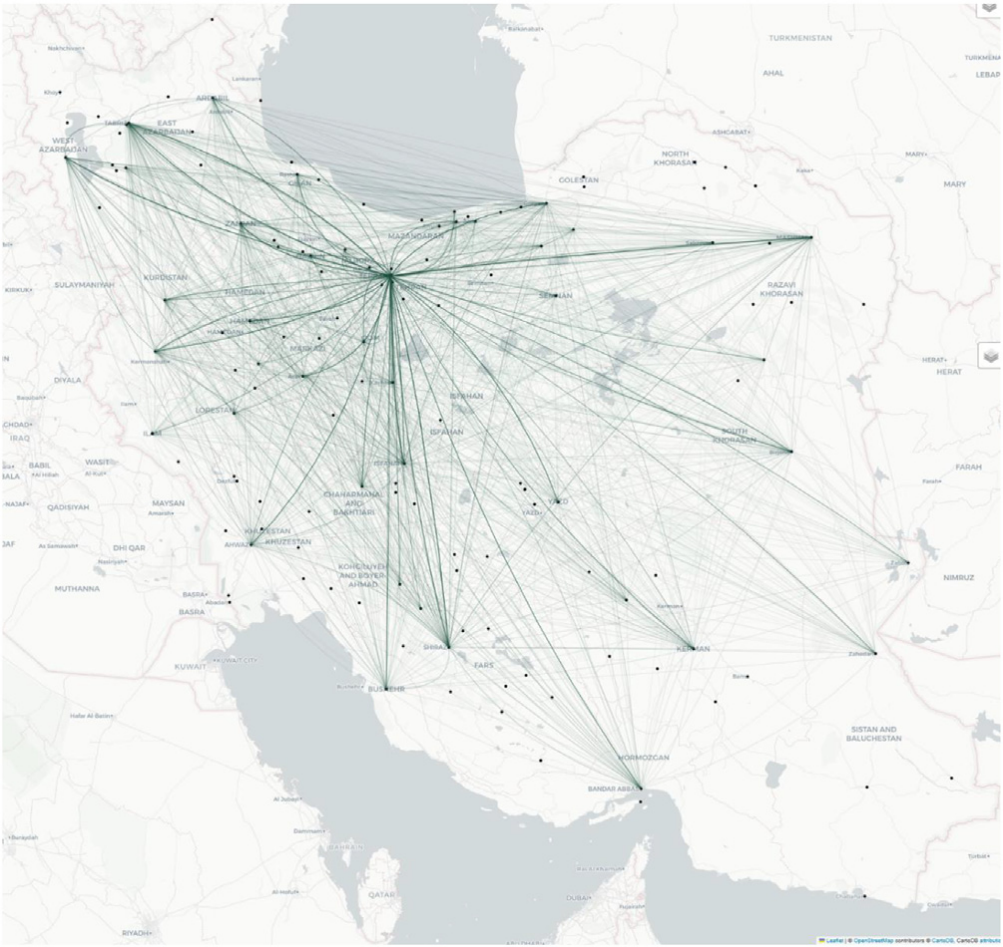
Collaboration network among Iranian universities based on co-authorship patterns.

Fig. 2 shows a map of collaboration among Iranian universities extracted from our dataset. Which forms “University Collaboration Networks” and help us identify which universities collaborate frequently by analysing co-authored publications.

Network mapping and co-authorship visualization were performed using tools that have become standard in bibliometric research [16]. We follow established practice from collaboration-network studies and recommend fractional (not just full) counting when projecting authorship to co-author edges; interactive inspection is supported in Gephi [17,18]. This collection facilitates comprehensive bibliometric research, institutional benchmarking, and academic network studies, making it a valuable resource for understanding and advancing academic ecosystems in Iran.

### 3.1 Data size

We have 4 files in total all in csv format. professors’ and universities’ dataset are naturally within professors and universities csv files. final_articles is a curated subset of articles.csv with more metadata (Table 1).

**Table 1:** Data files and corresponding number of records.

File	Records
Universities	90
Professors	∼50,000
Articles	∼1.5 million
final_articles	∼184′000

**1. Universities Dataset:**

The universities.csv file contains data on nearly every university in Iran, including national rankings, geographic details, and metadata for organizational analysis. University ranks were extracted from https://www.4icu.org/ir/. The CSV variables in order they appear in the CSV file are: “#Rank”, “University”, “Town”, “org_id”, “GNM”, “latitude” and “longitude”.

Table 2 shows what’s in this dataset:

**Table 2:** University Dataset.

Mnemonic	Description	Format	None Values
#Rank	National rank of the university	Int	0 %
University	Full name of the university	Str	0 %
Town	Town or city where the university is located	Str	0 %
org_id	Unique ID of the organization (Google Scholar)	Str	70 %
GNM	Is a university governmental and non-medical (True or None)	Bool	0 %
latitude	latitude of located city	Float	3 %
longitude	Longitude of located city	Float	3 %

Table 3 is a sneak peek at the dataset:

**Table 3:** University dataset sample.

#Rank	University	Town	org_id	GNM	latitude	longitude
1	University of Tehran	Tehran	3127,243,484,376,623,607	1	35.68925	51.3896
2	Sharif University of Technology	Tehran	14,542,101,698,899,415,237	1	35.68925	51.3896

**2. Professors Dataset:**

The professors.csv file contains detailed profiles of professors, showcasing their professional achievements and research impact. The data in this file has been updated as of the end of 2025, ensuring it reflects the most recent information available. Interest field is an optional field that some professors haven’t filled out in their Google Scholar profiles. Some cited_by_all values are none because selenium couldn’t fetch that professors’ citation data either because the professors google scholar URL was changed or deleted; note that zero isn’t considered as None Value, some professors have zero cited_by_all and this isn’t counted in None Values. The CSV variables in order they appear in the CSV file are: “id”, “name”, “university”, “user_id”, “affiliation”, “v_email_at”, “cited_by_all”, “cited_by_since_2020″, “h_index_all”, “h_index_since_2020″, “i10_index_all”, “i10_index_since_2020″ and “yearly_data”.

You can see the fields included in the dataset in Table 4:

**Table 4:** Professor Dataset.

Mnemonic	Description	Format	None values
id	Unique identifier for the professor	Int	0 %
name	Full name of the professor	Str	0 %
university	University affiliation of the professor	Str	0 %
user_id	Unique Google Scholar user ID	Str	0 %
affiliation	Professional title and department	Str	0 %
v_email_at	Verified email domain	Str	0 %
interests	List of primary research interests	Str	8 %
cited_by_all	Total number of citations received (all time)	Int	1 %
cited_by_since_2020	Citations received since 2020	Int	1 %
h_index_all	H-index over the entire career	Int	1 %
h_index_since_2020	H-index since 2020	Int	1 %
i10_index_all	i10-index over the entire career	Int	1 %
i10_index_since_2020	i10-index since 2020	Int	1 %
yearly_data	Yearly citation data in a JSON format	Dict	2 %

Table 5 shows the first record of the dataset:

**Table 5:** Professors' dataset sample.

Key	Value
id	0
name	Ali Fahim
university	University of Tehran
user_id	_C4Iif8AAAAJ
affiliation	Assistant Professor of Physics, University of Tehran
v_email_at	ut.ac.ir
interests	Elementary Particle Physics, Physics of Complex Systems, Data-Driven Sciences
cited_by_all	136,197
cited_by_since_2020	33,327
h_index_all	164
h_index_since_2020	76
i10_index_all	303
i10_index_since_2020	248
yearly_data	{'2010′: 526, '2011′: 2426, '2012′: 5812, '2013′: 8721, '2014′: 10,756, '2015′: 13,319, '2016′: 16,435, '2017′: 16,917, '2018′: 15,946, '2019′: 11,222, '2020′: 7744, '2021′: 6886, '2022′: 5972, '2023′: 6746, '2024′: 5941}
	

**3 Articles Dataset:**

The articles.csv file includes metadata for research publications, detailing titles, authorship, and citation metrics. More info isn’t available for all articles and “year” isn’t listed for all articles. Keep in mind that Article dataset isn’t processed, it’s the raw data extracted from Google Scholar. The CSV variables in order they appear in the CSV file are: “id”, “title”, “GS_link”, “year”, “cite”, “main_authors”, “more_info” and “link_ids”.

The layout of the dataset is shown in Table 6:

**Table 6:** Article Dataset.

Mnemonic	Description	Format	None Values
id	Unique identifier for the article	Int	0 %
title	Title of the article	Str	0 %
GS_link	Link to the article on Google Scholar	Str	0 %
year	Year of publication	%Y	6 %
cite	Number of citations received	Int	0 %
main_authors	List of main authors	Str	0 %
more_info	Additional publication details if mentioned (journal name, volume, year)	Str	7 %
link_ids	Dash (-) separated list of related professor’s ids	Str(‘-‘ sep)	0 %

You can see the first record of the dataset in Table 7:

**Table 7:** Article dataset sample.

Key	Value
id	0
title	Observation of a new boson at a mass of 125 GeV with the CMS experiment at the LHC
GS_link	/citations?view_op=view_citation&hl=en&user=_C4Iif8AAAAJ&citation_for_view=_C4Iif8AAAAJ:u5HHmVD_uO8C
year	2012
cite	22,285
main_authors	S Chatrchyan- V Khachatryan- AM Sirunyan- A Tumasyan- W Adam- …
more_info	Physics Letters B 716 (1)- 30–61- 2012
link_ids	0–5600–21,725–21,726–33,843–34,234

4 Final Articles Dataset:

The final_articles.csv file is a refined and processed version of the articles.csv file, retaining only:

4.1 Articles published between 2020 and 2022.

4.2 Articles linked to professors with >100 citations.

4.3 Articles associated with governmental and non-medical universities.

Additionally, articles with empty link_ids fields have been excluded, and the file includes extra information derived during the processing stage to enhance its utility. The CSV variables in order they appear in the CSV file are: “id”, “title”, “authors”, “publication_date”, “journal”, “volume”, “issue”, “pages”, “publisher”, “total_citations”, “description”, “GS_link”, “article_link”, “external_link”, “scholar_articles”, “citation_bars”, “link_ids” and “subject”.

Table 8 includes the dataset’s layout.

**Table 8:** Final Articles Dataset.

Mnemonic	Description	Format	None values
id	Unique identifier for the article	Int	0 %
title	Title of the article	Str	0.01 %
authors	List of authors	Str	0.09 %
publication_date	Date of publication	Y/(Y/M)/(Y/M/D)	0 %
journal	Name of the journal where the article was published	Str	13 %
volume	Volume of the journal	Int	13 %
issue	Issue number of the journal	Int	34 %
pages	Page range of the article	Str	11 %
publisher	Name of the publisher	Str	16 %
total_citations	Total number of citations received	Int	0 %
description	Brief description or abstract of the article (word limited abstract)	Str	4 %
GS_link	Link to the article on Google Scholar	Str	0 %
article_link	Main link of the article	Str	3 %
external_link	External links to supplementary materials or additional resources related to the article.	Str	48 %
scholar_articles	List of all versions of article in Google Scholar	List[Dict]	0 %
citation_bars	Yearly citation article received. in a JSON format	List[Dict]	30 %
subject	Academic subject or field. Assigned with GPT4o-mini API	Str	0.1 %
link_ids	Dash (-) separated list of related professor’s ids	Str(‘-‘ sep)	0 %

### 3.2 Derived multilayer representations and multi-path joins

We expose a heterogeneous graph with nodes U, P, A, S and layers: U–P (affiliation via professors.university ↔ universities.University), P–A (authorship via articles.link_ids ↔ professors.id), P–P (co-authorship by projecting P–A), and A–S (topics from final_articles.subject, aggregating to P–S and U–S). These joins also support top-down (U→P→A), bottom-up (A→P→U), and field-aware (…→S) paths. Exact column names and null rates are given in Table 2, Table 3, Table 4, Table 5, Table 6, Table 7, Table 8.

## 4 Experimental Design, Materials and Methods

We constructed a detailed dataset comprising approximately 1.5 million scholarly articles, each linked to its respective authors. This dataset serves as the foundation for our subsequent analysis, enabling a structured approach to evaluating academic performance and research trends. The multi-path join logic and multilayer graph schema are detailed in Data Description → Derived multilayer representations.

The universities serve as the foundational layer, containing data on approximately 90 universities across Iran. Initial profiles retrieved via Google Scholar included basic institutional information such as the university name and unique organizational ID. We scraped every Iranian university with a Google Scholar profile. Additional attributes like national rankings and geographic coordinates (latitude and longitude) were appended later during the data refinement process, enabling advanced spatial and organizational analyses. The dataset's initial focus on institutional affiliations laid the groundwork for comprehensive data integration in subsequent stages.

**Fig. 3:**
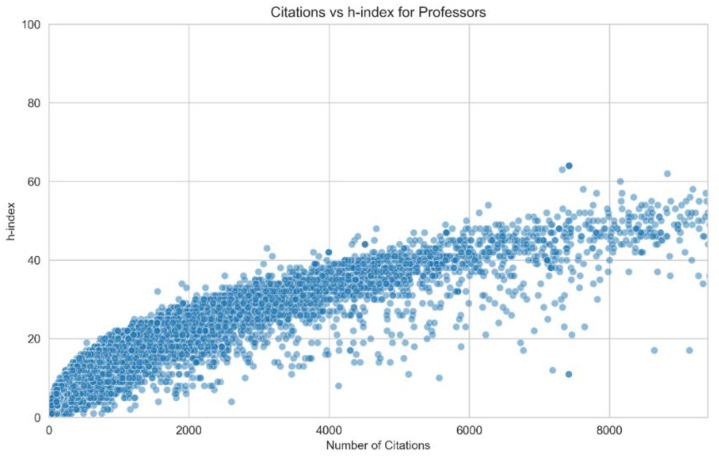
h-index per citation.

**Fig. 4:**
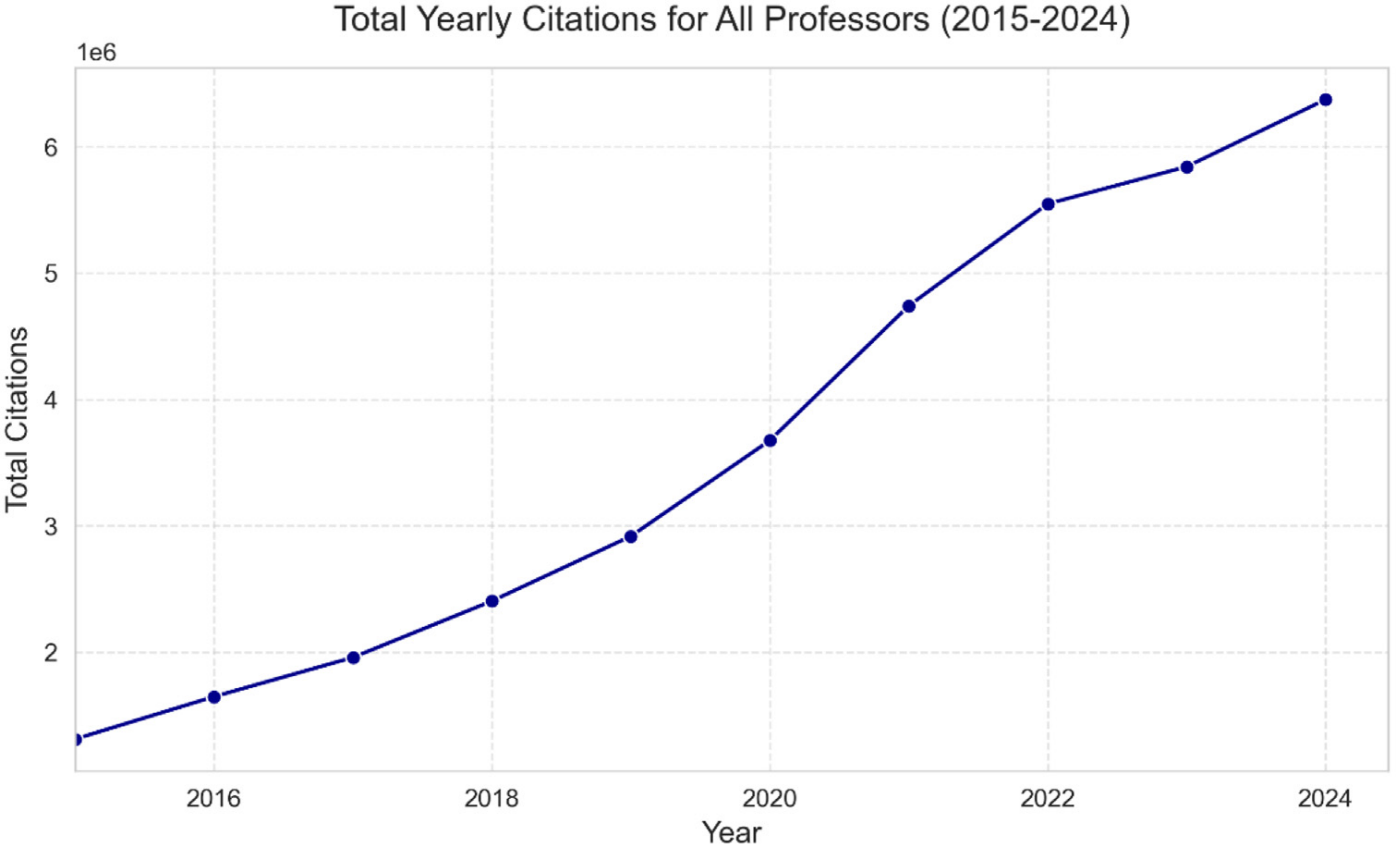
total citations per year.

The professors expand the dataset by profiling nearly 50,000 professors affiliated with these universities. Each record captures detailed metrics, including verified email domains, research interests, and citation data. Metrics such as the h-index, i10-index, and annual citation trends (as seen in Fig. 4) highlight the scholarly impact of individual professors. Fig. 3 shows h-index per number of citations for Iranian professors.

Data collection utilized linked profiles through Google Scholar's university pages, employing Python-based web scraping frameworks (Selenium and BeautifulSoup). Google Scholar’s coverage and citation data have been shown to align closely with other major bibliographic databases, making it a reliable source for large-scale scientometric studies [19]. These techniques ensured efficient navigation and comprehensive retrieval of profiles. Our scraping workflow adheres to best practices for automated data retrieval in Python [20].

**Fig. 5:**
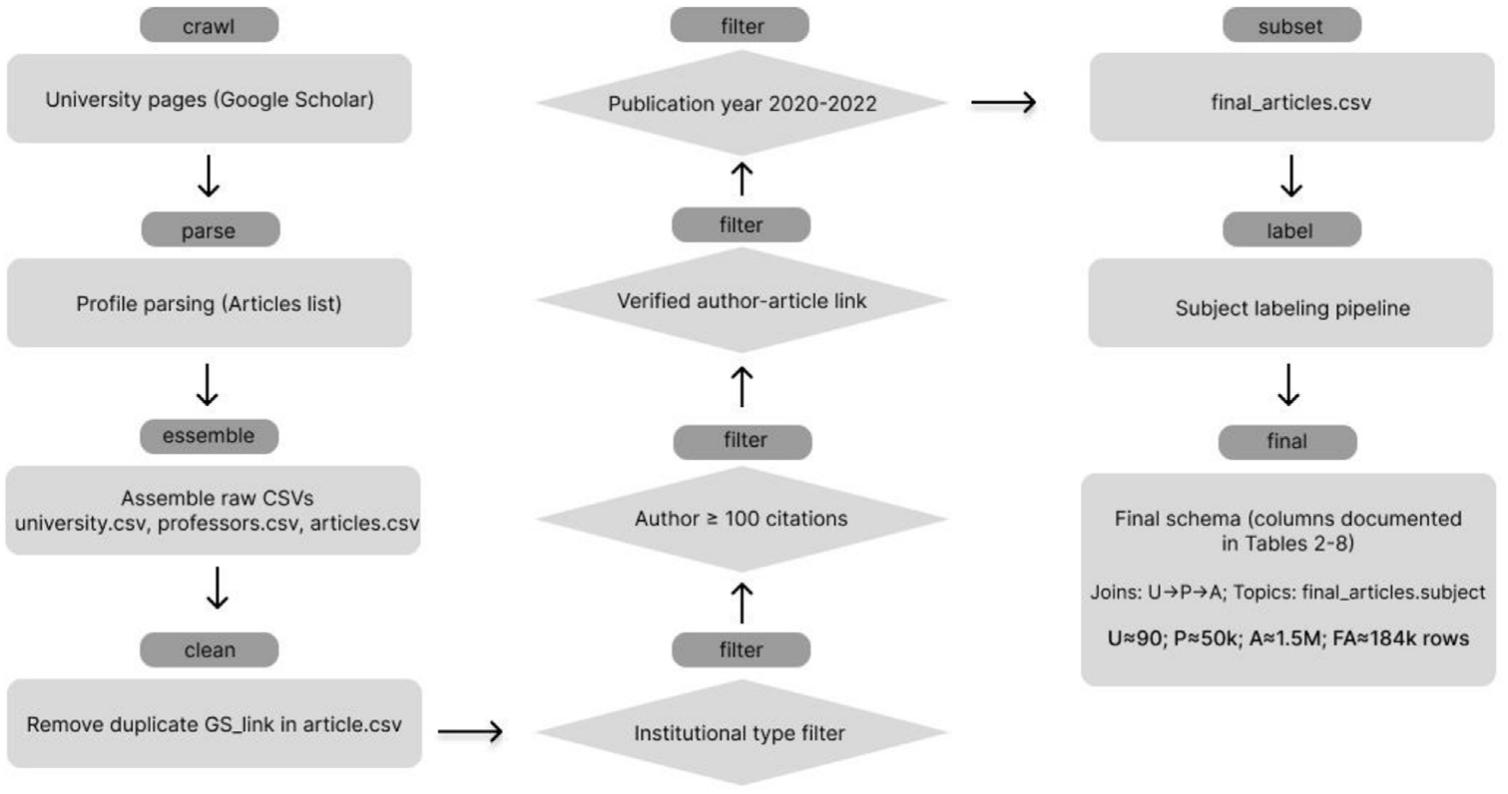
Harvest → Refinement → Enrichment pipeline.

The articles include metadata on approximately 1.5 million scholarly articles, capturing attributes like title, publication year, citation counts, and authorship details. Each article is uniquely linked to its respective professors via a shared identifier field. This dataset reflects a diverse range of academic outputs and serves as a foundation for analyzing research trends. Fig. 5 summarizes the harvest → refinement → enrichment pipeline, showing how the raw Google Scholar crawl of universities, professors and articles is progressively filtered and labeled to produce the curated, subject-annotated final_articles.csv subset.

### 4.1 Rationale for choosing google scholar and its reliability

**Table:** 

Criterion	Google Scholar (selected)	Scopus / Web of Science	Other open options (OpenAlex, free-tier Dimensions)
**Institutional access**	Freely reachable from any Iranian IP; scraping feasible with standard rate-limiting.	Our university holds **no subscriptions**; API and bulk download rights require paid licences [21,22].	Dimensions expose only throttled, partial APIs unless a fee is paid [23]. OpenAlex offers a generous free API; premium tiers add higher limits/frequency [24].
**Coverage of Iranian/Persian output**	Captures ≈ 90 % of citations found in Scopus/WoS and many unique ones [7]. GS indexes a markedly higher proportion of Persian-language journals and local conference papers than either Scopus or WoS [19].	Under-representation of small domestic titles; humanities most affected [25].	Still building Persian coverage; recent audits show discontinuities [26].
**Researcher-institution linkage**	Public author profiles plus “university” pages yield a directly crawlable **university ↔ author ↔ article** graph.	Author IDs and affiliations are behind paywalls; bulk affiliation data often incomplete.	-
**Licence for redistribution**	Zero licence fees → we can release the cleaned metadata under CC-BY. users should check downstream reuse conditions for third-party fields	Licence terms forbid open redistribution, undermining reproducibility.	Same restriction for Dimensions commercial tier.
**Scraping legality**	Viewed only publicly visible pages in a regular browser session with conservative pacing; no access controls were bypassed. Google services restrict automated access; we release derived metadata and advise users to ensure their own compliance.	No UI scraping performed. These services generally prohibit automated collection without explicit permission or a text-and-data-mining (TDM) agreement.	-

### 4.2 Reliability of google scholar data

#### 4.2.1 Strengths

• **High recall with stable relative metrics:** Multiple large-scale comparisons report that Google Scholar captures 95 % of Scopus citations and 92 % of WoS [7], and that scholar-level ranks (h-index, citation ordering) correlate at ***r* ≈ 0.88–0.93** with Scopus, even though absolute counts are higher [27].
• **Timeliness**: GS indexes “in-press” and repository versions months before they appear in subscription databases [24], which is critical for a 2020–2022 snapshot intended to inform current MSRT policy.

#### 4.2.2 Known weaknesses and our mitigations

**Table:** 

Issue in literature	Mitigation in this study
Sauvayre (2022) reports median error 14.6 % with ranges up to ∼53.5 % across studied corpora, and a separate small sample with extremely high error [28]	Exact-duplicate links were purged in articles.csv; all other metadata remain as scraped. Analytical filters were applied only in final_articles.csv (see Data Refinement Process).
Citation inflation and potential manipulation [29]	-
Incomplete affiliation strings	Only records linked through an official Google-Scholar university page were kept, ensuring a verifiable affiliation for every scholar.

Given (i) the absence of institutional access to Scopus or Web of Science, (ii) Scholar’s demonstrably superior coverage of Iranian and Persian-language research, and (iii) the cleaning and validation steps we applied to counteract its known metadata issues, Google Scholar is the only realistic, reproducible, and policy-relevant source for constructing an open scientometrics corpus on Iranian universities.

## 5 Methodology

### 5.1 Data sources and scope

We harvested data exclusively from Google Scholar using Python (Selenium + BeautifulSoup), traversing University pages → Professor profiles → Article lists. The crawl was throttled and distributed over ∼3 months to respect Google Scholar’s limits. We release four CSVs: universities.csv (∼90 rows), professors.csv (∼50,000), articles.csv (∼1.5 M), and a policy-oriented derivative final_articles.csv (∼184k) for worked examples.

• **Universities layer.** We scraped all Iranian universities with a Google Scholar profile, then appended “4icu” national rank and geographic coordinates to support spatial analyses. Columns: #Rank, University, Town, org_id, GNM (governmental & non-medical flag), latitude, longitude.
• **Professors layer.** From each university page we collected linked author profiles and metrics (e.g., cited_by_all, h_index_*, i10_index_*, yearly_data). The dataset reflects a 2024 crawl for universities.csv and articles.csv. We subsequently refreshed professors.csv in 2025; no other files were modified.
• **Articles layer (raw).** For every professor we captured article-list metadata available on their Scholar profile: id, title, GS_link, year, cite, main_authors, more_info, link_ids (dash-separated professor IDs). Aside from removing 12 exact duplicate GS_link rows, we preserved articles.csv as scraped (e.g., some missing or implausible year).

### 5.2 Data cleaning

**Duplicate Removal:** We identified and eliminated 12 article records in “articles.csv” file that shared the same Google Scholar link (GS_link), ensuring each entry in the dataset is unique. Aside from this the “articles.csv” is pretty raw.

We deliberately limited mandatory cleaning to exact-duplicate links so that articles.csv remains a faithful snapshot of what Google Scholar exposed at the time of harvest. More aggressive steps, such as deleting incomplete rows, standardizing journal names, or imputing missing years, can improve some analyses while simultaneously discarding data that other researchers may need. By shipping a raw file alongside a fully worked, policy-oriented derivative (final_articles.csv), we provide both (i) an untouched provenance layer and (ii) a blueprint that future users can customize to their own quality thresholds and research questions. The trade-offs of deeper cleansing are summarized in Table 9.

**Table 9:** Rationale for limiting mandatory cleaning to duplicate removal in articles.csv.

Why we stopped after removing exact duplicates	How the design choice is reflected in the paper
**Protects analytical versatility.** Dropping rows with missing fields, imputing years, or standardising journal titles would lock the dataset into our assumptions (e.g., which imputations are “acceptable”). Users who need those records for text mining, network-growth studies, or bespoke imputation strategies would lose potentially valuable information.	We explicitly declare that articles.csv is “raw data extracted from Google Scholar” and leave detailed filtering to downstream users.
**Preserves a verifiable provenance trail.** By releasing the crawl almost exactly as captured, including out-of-range years and incomplete metadata, we make it possible for others to audit our scrape, rerun it, or test alternative cleansing rules.	The manuscript distinguishes **Data Cleaning** (minimal) from **Data Refinement** (purpose-specific filters) and notes that the original files “were left unchanged as source files, preserving the complete dataset as it was initially collected with exclusion of duplicate records for *articles.csv*.”
**Avoids premature information loss.** Aggressive cleaning can inadvertently delete grey-literature items, early-online versions, or Persian-language records that lack standard bibliographic fields but are important for Iran-specific studies.	Limitations section flags remaining metadata gaps (e.g., 6 % missing years) and advises users to “impute or exclude … when time-sensitive indicators are computed.”
**Provides a worked example instead of a one-size-fits-all solution.** We illustrate how others can subset and enrich the raw file by publishing **final_articles.csv**, produced through transparent, reproducible steps (temporal window, citation threshold, institutional filter). Researchers can reuse or adapt that blueprint.	Section **Data Refinement Process** lists each filter step (institution type, > 100 citations, 2020–22 date range, link-ID check) applied only to create final_articles.csv; the raw source remained intact.
**Aligns with FAIR data principles.** Publishing both the raw corpus and a curated derivative satisfies the “Accessible” and “Reusable” pillars without compromising “Findability” or “Interoperability.”	The repository shares all four CSV files and documents variable-level null rates so users can decide how to proceed.

### 5.3 Data refinement process

To tailor the original dataset to our specific research objectives, we carried out a multi-step refinement process. This process was distinct from the original data compilation and did not alter the initial source dataset (articles.csv remains raw with exclusion of duplicate records. The following four filters were applied exclusively to a working copy, yielding final_articles.csv). Instead, the filtering was applied to create a refined version of the data, ensuring clarity, accuracy, and alignment with the study’s goals. To clarify provenance and the separation between raw harvest, curated subset, and enrichment, Fig. 5 summarizes the end-to-end data lineage, from Google Scholar university pages to the refined final_articles.csv and subject labeling.

Below, we outline the key filtering steps:

**1. Institutional Affiliation Filtering**

This step involved excluding records related to professors affiliated with non-governmental and medical universities. To achieve this, universities were manually classified as "governmental" or "non-governmental," ensuring accurate differentiation. Only data linked to professors from government-affiliated universities were retained in the refined version of the dataset. All records related to professors and their corresponding articles from non-governmental and medical universities were removed from the working dataset.

**2. Citation-Based Filtering**

To focus on high-impact researchers, we applied a minimum citation threshold. Professors with fewer than 100 citations were excluded from the refined dataset. This threshold ensured that only professors with a with a substantial citation profile remained in the analysis.

The exclusion process involved updating the 'link_ids' field in the *'final*_*articles.csv'* file, ensuring that the IDs of professors with fewer than 100 citations were removed. As a result, articles were removed only if all their authors were excluded. Articles with some excluded authors but retaining at least one included author were not removed.

**3. Article Association Verification**

A crucial aspect of the data refinement process was the verification of article-professor associations. Articles that lacked a corresponding 'link_id', an identifier linking them to a specific professor, were considered unassociated with any of the remaining professors. Such articles were removed to preserve data integrity, ensuring that each retained article could be traced back to a valid author within the revised dataset.

**4. Publication Date Range Specification**

**Fig. 6:**
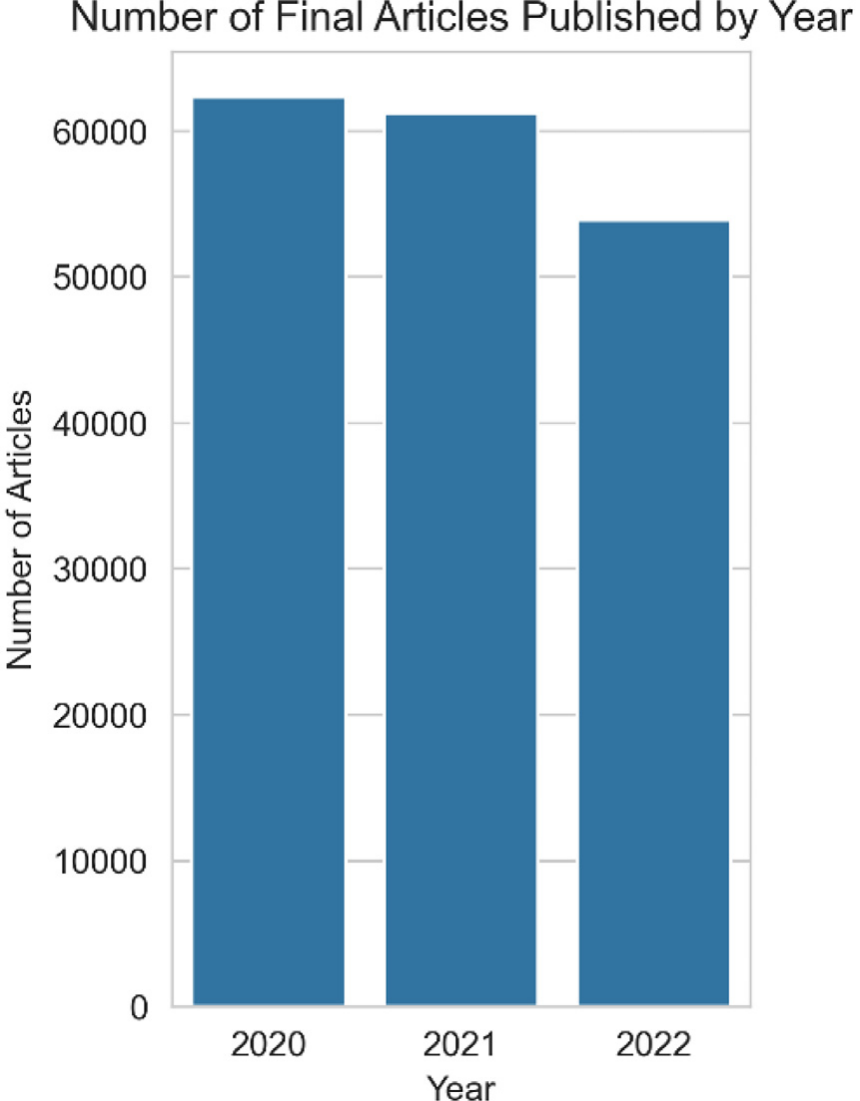
Number of articles published annually (2020–2022) in the final_articles dataset.

To ensure the dataset reflected recent and complete research outputs, we included only articles published between 2020 and 2022. This time frame provides a current perspective on academic contributions, enabling insights into contemporary research trends. In Fig. 6 you can see total number of articles published per year.

### 5.4 Enrichment of the refined subset

After the above filters, **∼184k** articles remained. For these only, we re-scraped by clicking into each article’s page on Google Scholar (Selenium + BeautifulSoup) to extract richer metadata now present in final_articles.csv (e.g., authors, publication_date, journal, volume, issue, pages, publisher, total_citations, description, article_link, external_link, scholar_articles, citation_bars, subject). This “click-in” enrichment explains why final_articles.csv carries additional fields beyond the profile-list fields in articles.csv.

### 5.5 Subject classification

**Input:** For each row in final_articles.csv, we supplied **Title + Description** to the classifier.

**Label set**: We used 23 FoR-style categories from ANZSRC/ERA 2020 [30] as the base set; after inference, three closely related fields (Biological Sciences, Health Sciences, Biomedical & Clinical Sciences) were merged into Life Sciences & Biomedicine, yielding 21 customized subjects (see Table 10. Key subject Areas, modified and customized version of FoR codes for the final list).

**Table 10:** Key subject Areas, modified and customized version of FoR.

Agricultural, Veterinary and Food Sciences	Life Sciences & Biomedicine	Physical Sciences
*Built Environment and Design*	*Chemical Sciences*	*Commerce, Management, Tourism and Services*
*Creative Arts and Writing*	*Earth Sciences*	*Economics*
*Education*	*Engineering*	*Environmental Sciences*
*Psychology*	*History, Heritage and Archaeology*	*Human Society*
*Indigenous Studies*	*Information and Computing Sciences*	*Language, Communication and Culture*
*Law and Legal Studies*	*Mathematical Sciences*	*Philosophy and Religious Studies*

### 5.6 Key subject areas (Customized and final)

**Model: ChatGPT 4o-mini** in **zero-shot** mode with temperature set to Zero.

**Fig. 7:**
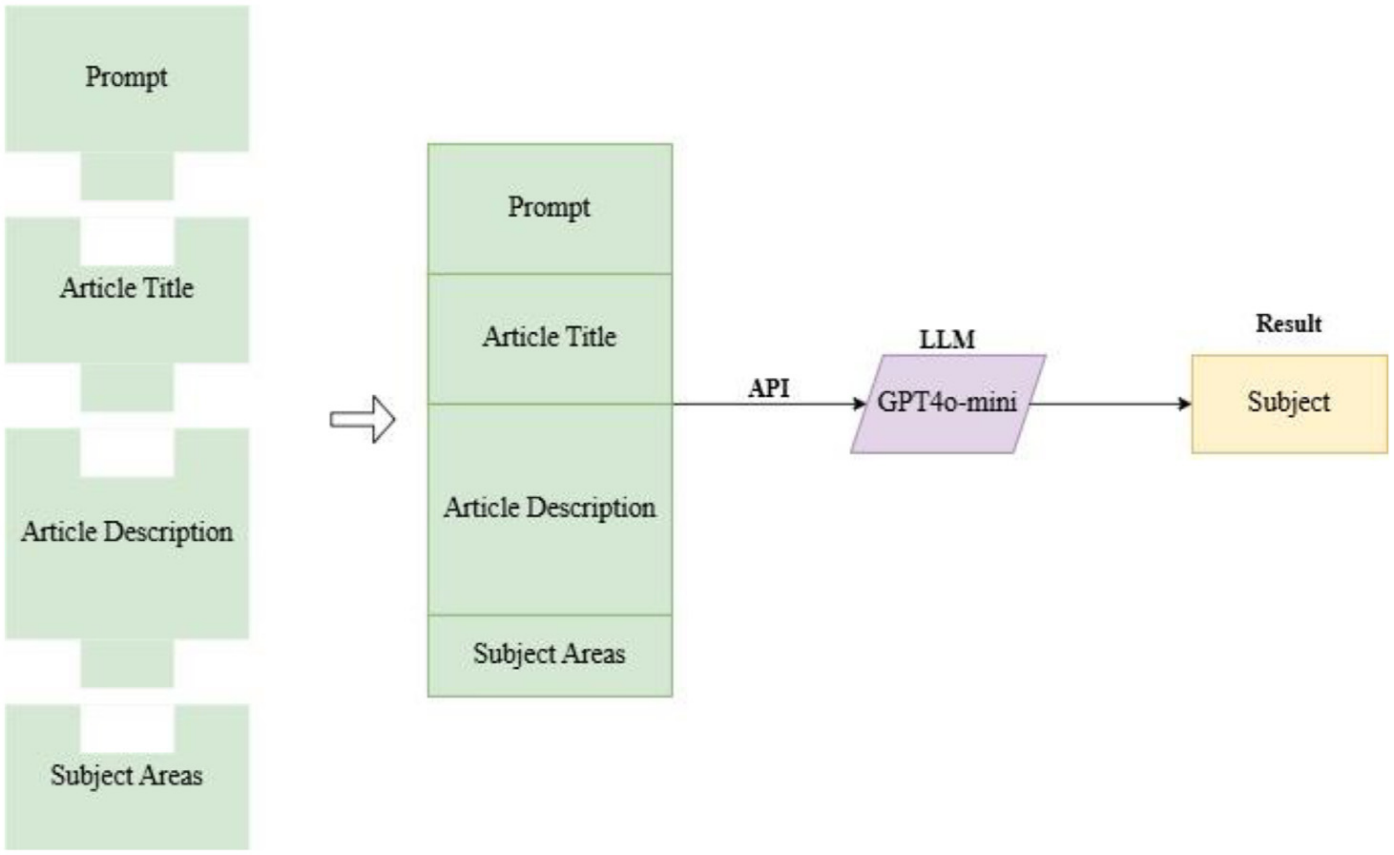
work flow for subject classification.

Fig. 7 shows the workflow for Subject classification used in this article.

**Code:** The initial scraping codes can be found in the following link: https://github.com/Mmli081/Articles-analysis **Prompt:** The model was instructed to choose exactly one label from **23 FoR-style categories Post-processing to 21 categories:** Following labeling, Biological Sciences, Health Sciences, and Biomedical & Clinical Sciences were merged into “Life Sciences & Biomedicine,” yielding 21 customized subject areas (Table 10).

**Fig. 8:**
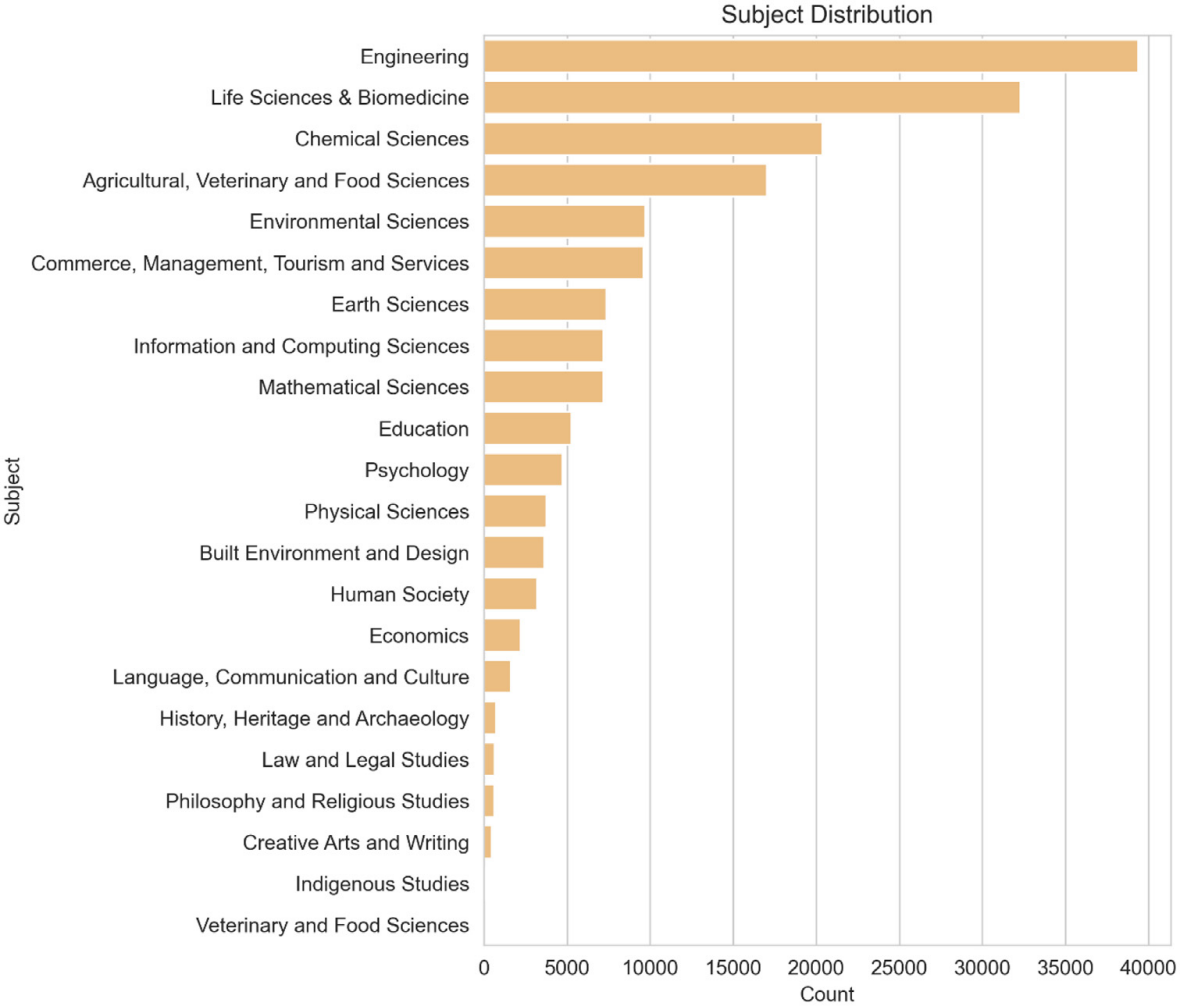
Subject distribution in Final Articles dataset.

**Subject Distribution:** After completing the classification task, we observed the subject distribution and the number of articles categorized within each subject area, as illustrated in Fig. 8.

**Expert review:** We evaluated subject labels using five PhD-level faculty whose expertise covers the FoR domains present in our sample. A stratified random sample drawn from final_articles.csv matched the corpus-level subject distribution. For inter-rater checks, 20 anchor articles were rated by all five experts (100 total judgements). Each packet included the article ID, title, a word-limited abstract, and the model-assigned label; reviewers confirmed correctness or disagreed.

Five domain experts served as validators: (1) Nosaibah Ebrahimi, Dept. of Chemistry, Faculty of Science; (2) Alireza Abdollahpouri, Associate Professor, Dept. of Computer, Faculty of Engineering; (3) Roonak Daghigh, Dept. of Mechanical Engineering, Faculty of Engineering; (4) Sholeh Ebrahimpour, Assistant Professor of Clinical Pharmacy; (5) Zeynab Aeeni, Faculty of Humanities and Social Sciences.

For compactness, we report the anchor IDs, the 20 IDs are: 76,929, 1304,750, 31,359, 1405,333, 83,250, 1474,336, 1099,035, 862,770, 1004,993, 656,253, 1403,092, 864,743, 207,482, 1437,830, 168,552, 73,291, 955,587, 1314,666, 144,329, 1455,749. Titles/abstracts are retrievable by ID in final_articles.csv

### 5.7 Notes for users (data integrity)

• **Years.** Raw articles.csv intentionally contains anomalies to preserve provenance: ∼6 % clearly incorrect years (e.g., 1, 15, 377), ∼6 % missing (None), and **4477** Shamsi years (1360–1402). Use imputation/exclusion as needed for time-normalized indicators.

### 5.8 Automated classification reliability

To anchor our zero-shot subject labeling in empirical evidence, we draw on OpenAI’s published evaluation of GPT-4o-mini across standard reasoning and classification benchmarks:

• MMLU (Massive Multitask Language Understanding): GPT-4o-mini achieves 82.0 % accuracy on a 57-subject multiple-choice exam spanning STEM, humanities, and social sciences, demonstrating strong multi-domain classification ability [31].
• MGSM (Multilingual Grade-School Math): Scoring 87.0 %, GPT-4o-mini outperforms earlier compact models on multilingual math reasoning tasks [31].
• HumanEval (Code Generation): With 87.2 % functional correctness on program-synthesis problems, it shows robust pattern recognition and syntax understanding [31].
• MMMU (Massive Multi-discipline Multimodal Understanding & Reasoning): At 59.4 %, GPT-4o-mini handles combined text-and-image reasoning, confirming its capacity for complex, multi-modal inputs [38]

Moreover, GPT-4o-mini’s performance on MGSM and HumanEval (both ≥87 %) indicates strong reasoning capabilities that could transfer to our domain of structured metadata (Table 11).

**Table 11:** ChatGPT 4o-mini Scores.

Benchmark	Task Description	GPT-4o-mini Score ( %)
MMLU	57-way multi-domain multiple-choice classification	82.0
MGSM	Grade-school math reasoning across languages	87.0
HumanEval	Functional correctness on code generation problems	87.2
MMMU	Multimodal (text + image) understanding & reasoning	59.4

## Limitations

Despite the breadth and granularity of the corpus, several constraints documented elsewhere in the manuscript necessarily bound its present utility:

1. **Source-level coverage and metadata gaps (Google Scholar):**
  All records were harvested exclusively from Google Scholar. While the platform offers the largest free index of Iranian research, its metadata are neither schema-controlled nor complete: for example, *org_id* is missing in 70 % of university rows, *external_link* in 48 % of final_articles, and *citation_bars* in 30 % of those same rows (see Tables 2, 8). These omissions may limit certain longitudinal or geospatial analyses until users supplement the data from external bibliographic services.
2. **Residual missing or uncertain publication years:**
  Because we preserved raw dates in articles.csv, roughly 6 % of entries show clearly incorrect years (e.g. 1, 15, 377), and about another 6 % feature a blank (None) year field and 4′477 (≈ 0.43 %) records specify a Shamsi year from 1360 to 1402. Users should impute or exclude these when computing time-normalized indicators.
3. **Selective cohort design in *final_articles.csv*:**
  The filtered subset retains only publications (i) dated 2020–2022, (ii) linked to professors with > 100 citations, and (iii) affiliated with governmental, non-medical universities (Data Refinement section). This design maximizes policy relevance for MSRT benchmarking but necessarily under-represents private campuses, medical universities, early-career scholars, and articles outside the 2020–2022 window. Findings generalized beyond that population should therefore be interpreted with caution.
4. **Automated subject classification accuracy:**
  Article-level field labels in *final_articles.csv* were assigned by a zero-shot GPT-4o-mini model. Misclassifications, especially for multidisciplinary titles or under-represented fields, may propagate error into field-normalized indicators unless users re-validate tags for critical subsets.
5. **Asynchronous citation snapshots:**
  The crawl was deliberately throttled and distributed over a three-month window to respect Google Scholar’s implicit rate limits. Citation counts for articles scraped early in the window may differ slightly from those captured later. Although the effect is minor for aggregate analyses, precision studies (e.g., month-by-month citation accrual) should account for this temporal skew.

By foregrounding these documented limitations, we aim to guide users toward appropriate secondary cleaning, enrichment, or validity checks before drawing high-stakes inferences from the dataset.

## Ethics Statement

We confirm that all authors have read and are in compliance with the ethical requirements for publication in Data in Brief. Additionally, we confirm that this work does not involve human subjects, animal experiments, or any data collected from social media platforms.

## Credit Author Statement

**Mohammad Shafei:** Conceptualization, Visualization, Validation, Software, Investigation, Formal analysis, Data Curation, Methodology. **Parsa Zahedi:** Validation, Software, Writing - Original Draft, Writing - Review & Editing, Formal analysis, Resources. **Rojiar Pirmohamadiani:** Supervision, Project administration, Conceptualization.

## Data Availability

Mendeley DataIranian Scientometrics; Dataset on Universities, Professors and Articles (Original data)
